# *CORR* Insights^®^: Report of the Clinical and Functional Primary Outcomes in Men of the ACL-SPORTS Trial: Similar Outcomes in Men Receiving Secondary Prevention With and Without Perturbation Training 1 and 2 Years After ACL Reconstruction

**DOI:** 10.1007/s11999-017-5309-6

**Published:** 2017-03-08

**Authors:** Stephanie R. Filbay

**Affiliations:** Arthritis Research UK Centre for Sport, Exercise & Osteoarthritis, Nuffield Department of Orthopaedics, Rheumatology & Musculoskeletal Sciences, University of Oxford, Botnar Research Centre, Windmill Rd, Oxford, OX3 7LD UK

## Where Are We Now?

Returning to sport is a key determinant of longer-term quality of life after ACL reconstruction [5]. Although most patients expect to return to preinjury sport after ACL reconstruction [4], only 60% of nonelite athletes fulfil this expectation [2]. Of further concern, one in four young athletes who return to sport suffer a graft rupture or contralateral ACL rupture [14]. Unfortunately, people who have a revision ACL reconstruction or rupture their contralateral ACL are likely to experience persistent knee difficulties and poor quality of life [6, 10].

It is possible that many rehabilitation programs are falling short in the later stages when it comes to physically and psychologically preparing an ACL reconstructed individual to return to sport. A primary aim of ACL rehabilitation is to restore physical knee deficits, yet restoration of knee deficits does not correspond to a successful return to sporting performance or prevention of further knee injury. Evidence is limited surrounding predictors of successful rehabilitation, return to sport, and reinjury after ACL reconstruction [12].

The study by Arundale and colleagues, explored the benefit of adding perturbation training to a high level rehabilitation program designed to facilitate return to preinjury sport and minimize reinjury rates. The addition of perturbation training did not improve outcomes in this specific sample of ACL reconstructed men.

## Where Do We Need To Go?

Individuals who achieve dynamic knee stability after ACL rupture through rehabilitation alone, can return to sport with similar longer-term outcomes as those who underwent ACL reconstruction [8, 9]. However, most studies reporting longer-term outcomes after nonoperative management of ACL rupture, poorly describe and rarely standardize rehabilitation strategies [7]. Consequently, expanding research in this area has potential to increase the proportion of patients successfully managed without ACL reconstruction.

Instead of seeking an ideal rehabilitation approach to improve outcomes for all ACL ruptured individuals, there is a need to identify common characteristics of patients who respond favorably to specific elements of ACL rehabilitation. This will help guide tailored rehabilitation recommendations, based on the physical and psychological characteristics of an individual with acute ACL injury.

Too often ACL rehabilitation overlooks psychological barriers to returning to sport, including psychological readiness, low self-efficacy, knee confidence, and reinjury fears [3, 13]. A greater emphasis on addressing psychological factors during rehabilitation is warranted and a psychological assessment should be performed prior to return to sport.

Additionally, the KOOS-quality-of-life subscale is not ideal for assessing quality of life after ACL injury and reconstruction. An individual who is aware of their knee, or who modifies their lifestyle because of their knee, will have an impaired KOOS-quality-of-life score even if these are not negatively impacting upon their life quality. The ACL-quality-of-life score may be a more appropriate measure of quality of life following ACL injury and reconstruction [11].

## How Do We Get There?

The rehabilitation journey should not end on return to sport. After returning to sport, the focus should shift to returning to preinjury performance, followed by a maintenance phase to reduce risk of further knee injury.

Future studies delivering standardized ACL rehabilitation to participants should assure that rehabilitation strategies are described in reproducible detail. This should be done for preoperative rehabilitation, postoperative rehabilitation, and management with rehabilitation alone. This would enable future data pooling and meta-analysis, and advance current knowledge in this field.

There is also a need for randomized controlled trials comparing the efficacy of different rehabilitation strategies within groups at risk of poor longer-term outcomes (including people with concomitant meniscus injury, high fear of reinjury, worse patient-reported knee status, and a previous ipsilateral or contralateral ACL rupture [1, 10]).

ACL rehabilitation approaches may evolve through trialling new and novel interventions that extend beyond current practices and draw upon the neuroscience and psychological literature to address the neurophysiological and psychological impacts of ACL injury and reconstruction.
